# Advances and Challenges in Drug Screening for Cancer Therapy: A Comprehensive Review

**DOI:** 10.3390/bioengineering12121315

**Published:** 2025-12-01

**Authors:** Shohei Motohashi, Eriko Katsuta, Daisuke Ban

**Affiliations:** Department of Hepatobiliary and Pancreatic Surgery, Graduate School of Medicine, Institute of Science Tokyo, Tokyo 113-8510, Japan; motohashi.shohei@tmd.ac.jp (S.M.); d-ban.msrg@tmd.ac.jp (D.B.)

**Keywords:** drug screening, artificial intelligence (AI), multi-omics integration, pharmacogenomics, patient-derived organoids (PDO), patient-derived xenografts (PDX), organ-on-a-chip, CRISPR screening, biomarker discovery, precision oncology

## Abstract

Cancer drug screening is shifting from low-predictive, reductionist assays to human-relevant, data-integrated platforms. This review synthesizes preclinical strategies using a unified lens—Principle, Advantages, Limitations, and Clinical Application—to enable like-for-like comparison. We first appraise traditional two-dimensional (2D) monolayers and animal models, noting scalability and historical utility alongside constrained translational fidelity. We then evaluate advanced systems—patient-derived organoids (PDOs), patient-derived xenografts (PDXs), and organ-on-a-chip—that better recapitulate architecture, microenvironmental cues, and pharmacodynamics (PD), yet face trade-offs in throughput, timelines, costs, and standardization. Functional genomic screens (CRISPR/RNAi) and large-scale pharmacogenomics are summarized as engines for mechanism-based target discovery and resistance mapping, while AI-enabled modeling supports response prediction, biomarker development, and rational combinations. Finally, we discuss trial designs (basket/umbrella), drug repurposing lessons, and regulatory momentum for new approach methodologies. Across platforms, we emphasize cross-model validation, dataset harmonization, and clinically anchored endpoints as prerequisites for real-world impact. We conclude with pragmatic guidance for matching screening modality to study goals, sample constraints, and decision timelines to accelerate precision oncology.

## 1. Introduction

Cancer remains one of the most formidable diseases to treat due to its remarkable heterogeneity, evolutionary adaptability, and complex interactions within the tumor microenvironment [1]. Even among patients with the same histological subtype, therapeutic responses vary widely, and overall response rates for many standard regimens remain unsatisfactory [2,3]. Traditional drug discovery has largely depended on a sequential, hypothesis-driven approach involving single-target identification followed by preclinical testing in cell monolayers and animal models. Although this paradigm has produced several pivotal therapeutic advances, it remains slow and costly. While approximately 10% of agents identified through preclinical drug-screening pipelines ultimately achieve clinical approval—a rate that is not exceptionally low by historical standards—the enormous financial burden and resource intensity associated with bringing a single successful drug to market indicate that there is still substantial room for improving the efficiency and predictive power of early-stage screening strategies [2,3].

A major contributor to this inefficiency is the limited translational relevance of conventional preclinical models. Two-dimensional (2D) monolayer cultures, while scalable and standardized, fail to capture the three-dimensional (3D) architecture, stromal interactions, and immune components of human tumors. Conversely, animal models are more physiologically relevant but are labor-intensive, expensive, and often poorly predictive of clinical outcomes [4]. These shortcomings have driven the development of advanced preclinical drug screening platforms designed to better reproduce the complexity of human cancers and improve translational fidelity [5,6].

Over the past two decades, cancer drug screening has undergone a fundamental transformation—from reductionist, target-based discovery to empirical, large-scale profiling across diverse molecular contexts. Landmark resources such as the NCI-60 panel, Cancer Cell Line Encyclopedia (CCLE), and Genomics of Drug Sensitivity in Cancer (GDSC) have systematically linked molecular features with pharmacological responses across hundreds of cancer cell lines [7,8,9,10]. More recently, patient-derived organoids (PDOs), patient-derived xenografts (PDXs), and organ-on-a-chip systems have emerged as innovative platforms that preserve patient-specific phenotypes in ex vivo and in vivo settings. In parallel, CRISPR-based functional genomics and multi-omics integration have enabled unbiased identification of druggable vulnerabilities and predictive biomarkers [11,12,13,14]. Furthermore, artificial intelligence (AI) and traditional in silico tools are increasingly utilized to prioritize compounds, model therapeutic responses, and generate testable hypotheses.

**Figure 1:**
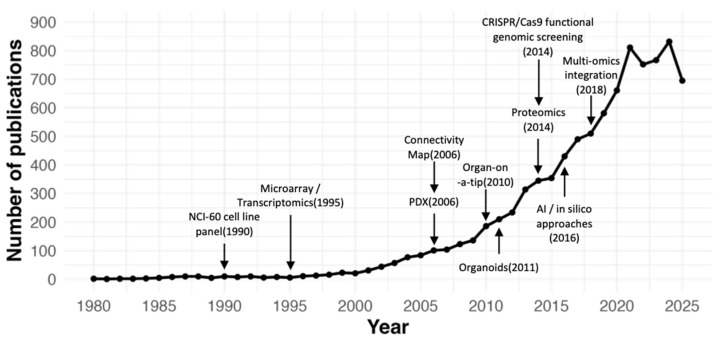
Annual number of publications on drug screening in the context of cancer research indexed in PubMed from 1980 to 2025. The search query combined drug screening–related terms with cancer-related terms. The figure shows a steady increase since the early 2000s, with a sharp rise after 2010, reflecting the expanding role of high-throughput drug screening in oncology.

This review provides a comprehensive overview of current drug screening strategies in oncology. We also highlight the translation of screening findings into basket and umbrella clinical trials, discuss the successes and failures of drug repositioning, and outline emerging directions including regulatory acceptance, ethical considerations, and integration with real-world clinical data. Notably, the rapid expansion of this field is reflected in the growing number of cancer drug screening-related publications indexed in PubMed over the past four decades, with a marked increase since 2010 (Figure 1).

**Figure 2:**
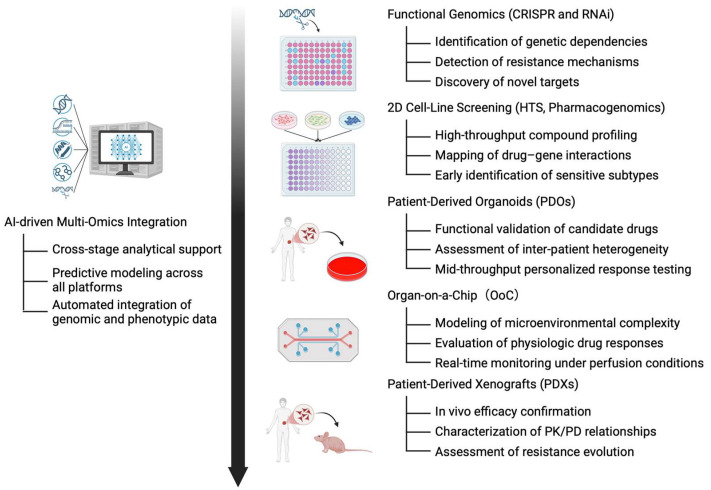
Integrated Workflow for Modern Preclinical Drug Screening. Functional genomics enables target discovery, 2D screening provides high-throughput compound profiling, PODs offer personalized validation, organ-on-a-chip systems model physiologic microenvironments, and PDXs confirm in vivo efficacy and PK(Pharmacokinetics)/PD. AI-driven multi-omics integration supports all stages with cross-platform analytics and predictive modeling.

As illustrated in Figure 2, modern drug-screening pipelines now combine functional genomics, 2D pharmacogenomic profiling, patient-derived models, microphysiological systems, and in vivo validation within a unified framework. While organ-on-a-chip platforms and PDX models appear sequential within this workflow, they in fact serve complementary and partially parallel roles—organ-on-a-chip systems providing microenvironment-level physiological insight, and PDXs offering whole-organism validation. The following sections examine each of these approaches individually, outlining their principles, advantages, and limitations.

## 2. Traditional Preclinical Screening Models

### 2D Cell Line Screens (Monolayer Culture)

Principle. The earliest systematic drug screening efforts in oncology were performed using immortalized cancer cell lines maintained in 2D monolayer cultures. Compounds were typically applied at graded concentrations, and pharmacological parameters such as IC_50_, EC_50_, or area under the curve (AUC) were calculated to quantify potency. A seminal initiative in this field was the NCI-60 panel, established in the late 1980s, which profiled 60 human cancer cell lines representing nine tumor types [7]. This effort provided the first systematic framework linking molecular phenotypes to drug sensitivity and revealed substantial heterogeneity in drug responses among cancer types [15]. Later, the advent of high-throughput molecular profiling enabled the creation of large pharmacogenomic datasets such as the Cancer Cell Line Encyclopedia (CCLE) and the Genomics of Drug Sensitivity in Cancer (GDSC) [7,8,9,10]. These resources comprehensively cataloged genomic, transcriptomic, and epigenomic features across hundreds of cell lines, coupled with response data for diverse compounds, and they remain fundamental for biomarker discovery and computational modeling.

Advantages. Despite the emergence of more physiologically relevant models, 2D monolayer cultures remain indispensable tools in drug discovery. Their major strength lies in scalability and throughput, which enables rapid and cost-effective screening of thousands of compounds. Standardized and easily reproducible culture conditions facilitate inter-laboratory comparability, while compatibility with robotic handling allows full automation for industrial-scale screening. Another key advantage is the depth of molecular annotation: these panels are extensively characterized at the genomic, transcriptomic, and proteomic levels, providing rich metadata for integrative analyses [7,8,9,10]. In addition, pharmacogenomic data from 2D cultures serve as critical training sets for machine learning models that predict drug sensitivity, forming a bridge between experimental and computational drug discovery.

Limitations. They lack the tumor microenvironment, and therefore fail to capture stromal support, extracellular matrix composition, and immune components that critically influence drug responses [16]. Serial passaging also promotes clonal selection, reducing intratumoral heterogeneity and generating cell populations that differ substantially from the original tumor [7]. Even at the technical level, assay variability is a recurring issue; different viability assays, such as ATP-based luminescence versus dye-exclusion tests, sometimes yield inconsistent results [17]. Most importantly, the predictive power of 2D assays for clinical efficacy remains modest, limiting their value as standalone preclinical decision tools [18,19,20].

Clinical Application. Although 2D monolayer screening is inadequate for direct clinical translation, it continues to play an essential role at the early stage of drug development. It remains the standard platform for initial hit discovery, enabling identification of cytotoxic or cytostatic compounds for further validation. Furthermore, pharmacogenomic resources such as CCLE and GDSC provide an enduring foundation for biomarker discovery and hypothesis generation. In modern translational pipelines, results obtained from 2D screens are often validated using more physiologically relevant systems—such as PDOs, PDXs, or organ-on-chip models—to improve predictive accuracy and translational fidelity [7,8,9,10,18,19].

## 3. Advanced Preclinical Models for Cancer Drug Screening

To provide an overview of how these models differ in scalability, physiological relevance, and clinical predictivity, their key characteristics are summarized in Table 1 before each platform is discussed in detail.

### 3.1. Patient-Derived Organoid (PDO)

Principle. PDO is three-dimensional (3D) cultures generated from patient tumor samples. This structure self-organizes into miniature versions of the original tumor and retains key histological features, genomic alterations, and transcriptomic profiles of the parental tissue [21,22]. Unlike conventional cell line, PDO preserve clonal heterogeneity, tumor-specific driver mutations, and differentiation gradients, thereby providing a highly representative model for personalized drug testing.

Advantages. PDO offers several advantages that are particularly valuable in translational research. It maintains the genetic fidelity of the source tumor, preserving oncogenic mutations and subclonal diversity that reflect patient-specific heterogeneity [21,22]. Their 3D architecture allows for physiological features such as cellular polarity, extracellular matrix interactions, and oxygen and nutrient gradients, which enhance biological relevance compared with two-dimensional cultures [21]. Although PDO systems are not yet truly high-throughput, they can be scaled to test dozens or even hundreds of compounds per patient, including drug–drug combinations [23]. Furthermore, PDO can be cryopreserved, enabling the creation of living biobanks that facilitate retrospective testing and population-level studies [22,23]. From an ethical and practical standpoint, PDO also contributes to the 3Rs principle—Replacement, Reduction, and Refinement—by decreasing reliance on animal experiments [23].

Limitations. PDO model have several limitations that constrain their capacity to fully reproduce in vivo tumor complexity. Standard PDO culture generally lacks stromal fibroblasts, immune cells, and vascular structures, and thus cannot completely recapitulate the tumor microenvironment or model tumor–immune interactions [24]. In addition, PDOs do not reproduce systemic pharmacokinetic processes such as absorption, distribution, metabolism, and excretion. Establishing organoid can be technically challenging, as success rates vary by tumor type and sample quality, with a tendency to favor aggressive or fast-growing clones [25]. The time required for organoid establishment and screening can also exceed the therapeutic decision window in rapidly progressing cancers, such as pancreatic ductal adenocarcinoma.

Clinical Application. Accumulating evidence supports the clinical utility of PDO as predictive tools for therapeutic response. A landmark study in gastrointestinal cancer demonstrated that PDO drug responses predicted patient benefit with 100% sensitivity and 93% specificity [21]. Meta-analyses across tumor types have confirmed the predictive validity of PDO-based drug testing [23], while ongoing prospective trials such as APOLLO are evaluating the feasibility of real-time PDO-guided treatment selection. Moreover, efforts are underway to integrate autologous immune cells into PDO cultures to enable assessment of immunotherapy responsiveness, opening new possibilities for functional immune-oncology testing [24].

### 3.2. Patient-Derived Xenograft (PDX)

Principle. PDX is established by implanting freshly resected tumor fragments from patients into immunodeficient mouse [25]. It retains the histological architecture, genetic heterogeneity, and growth kinetics of the original tumor across multiple passages, thereby providing one of the most clinically predictive platforms in preclinical oncology research. Because PDX recapitulates human tumor behavior within an in vivo microenvironment, it has become an indispensable tool for translational cancer studies.

Advantages. The principal advantage of PDX lies in its capacity to preserve the in vivo tumor context. Once engrafted, tumor undergoes vascularization and interacts with host stromal components, enabling more physiologically relevant pharmacokinetic and pharmacodynamic profiling [25]. Numerous studies have demonstrated strong concordance between drug response observed in PDX and clinical outcome in corresponding patients, underscoring their high predictive validity [26,27]. Moreover, PDX serves as valuable tool for elucidating mechanisms of acquired resistance under sustained therapeutic pressure, allowing researchers to track clonal evolution and adaptation [28]. These characteristics have led to the emergence of co-clinical trial designs, in which PDX experiments are conducted in parallel with ongoing human clinical trials to identify predictive biomarkers and optimize therapeutic regimens. Since PDX models cannot fully recapitulate the human tumor microenvironment owing to the absence of human immune and stromal components, complementary analyses using liquid biopsy-based approaches such as circulating tumor DNA (ctDNA) and circulating tumor cell (CTC) profiling have been incorporated to enable more comprehensive and dynamic assessment of tumor evolution [27].

Limitations. Despite its translational strength, PDX model presents several practical and biological limitations. It is inherently low-throughput, as each model requires extensive resources, space, and time for establishment and maintenance. Generating and expanding a PDX cohort can take several months, which precludes its use for real-time clinical decision-making [25]. Furthermore, tumor may undergo evolutionary drift and clonal selection during serial passaging, resulting in divergence from the original patient tumor [27]. The lack of a functional immune system in conventional PDX also limits their application for immunotherapy research, unless humanized mouse model incorporating human immune components are employed. In addition, aggressive tumor tends to engraft more successfully, introducing bias in favor of highly proliferative or metastatic phenotype [25].

Clinical Application. PDX is widely regarded as the gold standard for confirmatory preclinical testing of candidate therapeutics. Co-clinical trials have demonstrated their value in mirroring patient responses, elucidating resistance mechanisms, and validating biomarkers identified through other model systems [26,27,28]. More recently, the development of humanized PDX—created by co-engrafting human immune cells with tumor tissues—has expanded their use into the field of immuno-oncology, enabling assessment of immune checkpoint inhibitors and other immunotherapeutic strategies under controlled yet physiologically relevant conditions. Table 2 shows reported sensitivity, specificity, and overall concordance of PDO and PDX models across major tumor types. Although the available data are heterogeneous and derived from studies with variable methodologies, several tumor types—notably colorectal cancer, pancreatic cancer, and ovarian cancer—consistently demonstrate relatively high predictive accuracy for both PDOs and PDXs. These findings suggest that, despite inherent variability and limited sample sizes in certain indications, PDO/PDX platforms can achieve strong translational predictivity in selected tumor contexts.

### 3.3. Organ-on-a-Chip Systems

Principle. Organ-on-a-chip platform employs microfluidic device that culture tumor cells together with stromal, endothelial, or immune components under continuous perfusion [27]. This microsystem reproduces essential biophysical forces—including fluid shear stress, oxygen, and nutrient gradients—and enable real-time monitoring of tumor behavior during drug exposure. By integrating microenvironmental cues absent in conventional static cultures, organ-on-a-chip model aims to bridge the gap between in vitro assays and in vivo physiology.

Advantages. A major strength of organ-on-a-chip technology is its ability to reproduce microenvironmental realism. Through inclusion of stromal and vascular compartments, these chips capture aspects of drug delivery barriers and cellular crosstalk that are difficult to model in PDOs [27,44]. In addition, multi-organ coupling designs allow the linkage of tumor compartments with liver or gut modules to approximate systemic PK and toxicity [45]. Because the culture chambers require only small amounts of biopsy tissue, organ-on-a-chip models are suitable for studies involving rare or limited clinical specimens [46]. Another distinctive feature is their capacity for real-time functional readouts: integrated sensors and imaging modules enable dynamic measurement of cell proliferation, apoptosis, barrier integrity, and cytokine release under drug treatment [44]. Taken together, these properties are powerful complements to organoid and xenograft models in functional precision oncology.

Limitations. Most devices are custom-fabricated and operate at relatively low throughput, which restricts the suitability for primary large-scale drug screening [27]. Technical complexity is another barrier, as specialized microfluidic expertise and instrumentation are required for routine operation [45]. Although stromal and immune components can be incorporated, systemic physiological factors—such as endocrine or metabolic influences—remain absent unless multi-organ configurations are implemented [27,44]. Furthermore, while proof-of-concept results are encouraging, rigorous clinical validation is still limited, and inter-laboratory variability continues to hinder standardization and regulatory acceptance [27].

Clinical Application. Emerging studies have demonstrated the translational potential of organ-on-a-chip technology. Proof-of-concept investigations have shown that these platforms can predict neoadjuvant chemotherapy responses within clinically relevant timelines of approximately two to three weeks. In esophageal cancer, organ-on-a-chip systems successfully distinguished chemotherapy responders from non-responders, outperforming static PDO cultures [27]. Immune-enhanced chips co-culturing tumor cells with autologous immune populations are now being developed to evaluate immunotherapy responsiveness [47]. Beyond gastrointestinal and esophageal cancers, organ-on-a-chip approach has also been applied to breast cancer model. For example, Avula et al. demonstrated that breast cancer-on-chip platforms can reproduce tumor–immune interactions and predict chemotherapy responses more accurately than static cultures, highlighting their potential to advance functional precision oncology [46]. Collectively, these systems complement PDO and PDX by validating top drug hits under dynamic, physiologically relevant conditions.

In summary, across 2D cell lines, PDOs, PDXs, and organ-on-a-chip systems, each model offers distinct strengths and limitations when evaluated in terms of scalability, physiological relevance, and translational predictivity. Two-dimensional cell-line screens enable rapid, low-cost, high-throughput discovery but lack microenvironmental and immune context. PDOs preserve patient-specific heterogeneity and offer substantially higher predictive accuracy, yet remain limited by establishment time and incomplete microenvironmental representation. PDXs provide the most physiologically faithful in vivo context and serve as the gold standard for mechanistic and confirmatory studies, though their cost and long timelines preclude routine clinical deployment. Organ-on-a-chip platforms occupy an intermediate position, capturing dynamic microenvironmental cues and enabling real-time functional readouts, with emerging evidence of strong short-term predictive performance, but still requiring standardization and broader clinical validation. Collectively, these comparisons demonstrate that no single platform is universally optimal; instead, each contributes uniquely within a stage-specific, integrated translational pipeline—from early high-throughput discovery (2D) to personalized functional testing (PDOs), in vivo validation (PDXs), and microenvironment-aware assessment (organ-on-chip).

## 4. Functional Genomic Screening (CRISPR and RNAi)

Functional genomic screening plays a complementary role by identifying genetic determinants that modulate drug sensitivity and resistance. In contrast to chemical compound screening, CRISPR- and RNAi-based approaches systematically perturb genes across the genome to reveal causal relationships between genotype and drug response.

Principle. Early approaches based on RNA interference (RNAi) enabled partial knockdowns across thousands of genes, providing important biological insights but suffering from off-target effects and incomplete silencing that limited precision. The advent of CRISPR/Cas9 technology revolutionized this field by enabling highly specific gene knockout, repression (CRISPRi), or activation (CRISPRa) with unprecedented efficiency [11,12]. CRISPR- and RNAi-based screening platforms can be deployed in pooled or arrayed formats to identify essential genes, synthetic lethal interactions, or mechanisms of acquired resistance under therapeutic pressure. Typically, large libraries of small guide RNAs (sgRNAs) or short hairpin RNAs (shRNAs) are used to perturb genes across the genome—or within focused gene sets—and changes in cell viability or phenotype are quantified following drug exposure. Under drug selection, negative-selection (dropout) screens identify sensitizer genes whose loss increases drug sensitivity, whereas positive-selection (enrichment) screens detect resistance genes whose knockout enables cells to survive treatment [48]. For example, pooled CRISPR knockout screens under chemotherapy have systematically identified genetic drivers of chemoresistance by comparing sgRNA abundance between drug-treated and untreated conditions [49]. Zhong et al. further expanded this approach by screening 30 genome-wide libraries across seven chemotherapeutic agents, revealing both known and novel resistance genes, including the identification of PLK4 as a druggable vulnerability that overcomes oxaliplatin resistance [49]. Beyond single-gene interrogation, combinatorial CRISPR strategies enable the mapping of synthetic lethal interactions—gene pairs whose simultaneous loss leads to selective cell death. The SCHEMATIC framework, for instance, systematically performed pairwise knockouts across multiple tumor lineages, identifying thousands of synthetic lethal interactions and uncovering context-specific drug–gene dependencies [50]. In practice, researchers often perform a two-stage workflow: an initial genome-wide single-gene screen to identify resistance or sensitizer hits, followed by secondary combinatorial screens to pinpoint synergistic interactions that can inform rational drug combination strategies [51,52]. More recently, functional genomic screening has been coupled with single-cell transcriptomics to link genetic perturbations with phenotypic and molecular outcomes. Technologies such as Perturb-seq integrate pooled CRISPR libraries with single-cell RNA sequencing, allowing direct correlation between genotype, drug perturbation, and transcriptional response [53]. This approach not only identifies resistance genes but also elucidates transcriptional reprogramming events that drive drug escape. Taken together, these methodological advances have made functional genomic screening an essential component of modern drug discovery and translational oncology.

Advantages. The technology provides superior specificity and efficiency, minimizing off-target effects and producing more reliable loss-of-function phenotypes. In addition to knockdown studies, CRISPRi and CRISPRa allow controlled repression or activation of target genes, thereby expanding the range of functional analyses. Integration of functional genomic screening with transcriptomic and proteomic datasets further reveals downstream pathways and druggable vulnerabilities, providing mechanistic insight into how gene perturbations alter drug responses. Moreover, pooled CRISPR screens can now be combined with high-content single-cell readouts, such as Perturb-seq, to directly connect genetic perturbations with phenotypic and molecular responses at single-cell resolution [53].

Limitations. Despite its transformative potential, functional genomic screening presents several challenges. Results are often model-dependent, and gene–drug interactions identified in one cellular context may not generalize to others [12]. Knockout of essential genes can obscure relevant drug interactions, while the success of a screen depends heavily on careful library design, sufficient coverage, and robust bioinformatic analysis [54]. Furthermore, validation of candidate hits remains a substantial burden, requiring orthogonal confirmation in independent models and experimental systems. These practical and biological limitations underscore the importance of rigorous follow-up and cross-platform validation to ensure translational relevance.

Clinical Application. Functional genomic screens have provided key insights into mechanisms of therapeutic sensitivity and resistance in cancer. CRISPR-based studies have identified genetic alterations that confer sensitivity to PARP inhibitors—particularly in BRCA-related DNA damage repair pathways—and resistance to topoisomerase inhibitors [55]. One study revealed that PARP1 acts as a sensitizer to topotecan, PRKDC modulates doxorubicin response, and BCL2L1 contributes to multi-drug resistance [56]. These discoveries not only highlight the power of functional genomic screening to uncover actionable targets but also inform rational combination strategies, biomarker development, and drug repurposing efforts. Integration of screening results with large-scale pharmacogenomic datasets continues to accelerate the translation of genetic insights into precision oncology, bridging experimental findings and clinical application.

## 5. Large-Scale Pharmacogenomic and Multi-Omics Integration

Principle. Large-scale pharmacogenomic initiatives aim to integrate multi-omics datasets—encompassing genomics, transcriptomics, proteomics, and metabolomics—with systematic drug response profiles to identify predictive biomarkers and therapeutic vulnerabilities. Foundational resources in this domain include the Cancer Cell Line Encyclopedia (CCLE) and the Genomics of Drug Sensitivity in Cancer (GDSC), which catalog multi-omics and pharmacologic profiles across hundreds of human cancer cell lines [7,8]. The Dependency Map (DepMap) extends these datasets by incorporating CRISPR-based gene essentiality screens, enabling joint analyses of genetic dependencies and drug sensitivities [14]. Additional large-scale efforts such as the LINCS/Connectivity Map (CMap) have generated over one million transcriptomic profiles describing cellular responses to chemical and genetic perturbations, thereby enabling disease–drug signature matching and mechanism-of-action inference [57]. The Profiling Relative Inhibition Simultaneously in Mixtures (PRISM) platform, which employs barcoded multiplexed viability assays, has screened approximately 4500 compounds across 578 cell lines and revealed unanticipated anticancer activities among FDA-approved non-oncology drugs [13]. These resources collectively establish the foundation for a data-driven approach to pharmacogenomics that connects molecular context to therapeutic outcome. Proteomic and functional layers further enhance this framework. Proteomic atlases quantify protein abundance across large cell-line panels, offering pathway-level insight that often exceeds the predictive power of RNA-based models. For instance, the CCLE proteome and a pan-cancer proteomic map encompassing 949 cell lines revealed protein-level biomarkers that improved drug response prediction and clarified mechanism-specific dependencies [8,58]. In parallel, functional genomic datasets derived from CRISPR or RNAi dependency maps provide causal anchors that align pathway vulnerabilities with pharmacologic profiles, bridging correlative associations with mechanistic interpretation [59]. Together, these integrated efforts define the principle of large-scale pharmacogenomics: to synthesize orthogonal molecular, functional, and perturbational evidence into mechanism-aware predictors that can be calibrated from preclinical cell lines to clinical patient cohorts [60,61,62].

Advantages. Multi-omics integration offers several major advantages over single-layer pharmacogenomic analyses. By combining diverse omics modalities, researchers achieve a more comprehensive, systems-level view of drug response mechanisms [7,8,57,63,64]. This multidimensional approach enhances biomarker discovery, facilitating the stratification of patients likely to respond or resist therapy [63], and supports drug repurposing by linking disease-associated expression signatures with compound-induced perturbations [57,64]. Cross-validation across modalities—such as the inclusion of proteomic data in models trained on transcriptomic features—improves predictive robustness and reduces noise introduced by mRNA variability. Proteome-scale datasets, including those spanning 949 cell lines, have demonstrated that protein abundance and stoichiometry can outperform RNA-based predictors for certain drug classes [8,58]. Similarly, perturbational signatures derived from LINCS/CMap provide mechanistic context for repositioning and combination discovery, while PRISM’s pooled drug screening format efficiently uncovers lineage-restricted sensitivities with interpretable molecular correlates [13,57]. Functional genomics data further strengthens causal inference by linking dependency maps to pharmacologic response profiles. Integrating CRISPR or RNAi dependency data helps distinguish mere correlates from causal vulnerabilities, nominating candidate synthetic-lethal pairs for further validation [59]. Importantly, models trained on large pharmacogenomic panels can be transferred to patient-derived cohorts (e.g., TCGA) or organoid datasets after harmonization, establishing a bridge from preclinical discovery to clinical stratification [61,62]. Collectively, multi-omics integration enhances signal-to-noise ratio, improves biological interpretability, and enables predictive models with direct translational potential.

Limitations. Despite its power, large-scale pharmacogenomic integration faces several technical and conceptual challenges. Multi-omics correlations do not necessarily imply causality, and associations identified in cell lines may fail to translate to in vivo settings due to differences in microenvironmental context, clonal architecture, or tissue physiology. Batch effects, variability in experimental protocols, and differences in sequencing platforms can confound data integration, while cross-dataset discordance remains a persistent issue. Notably, seminal analyses revealed discrepancies in drug sensitivity measurements between CCLE and GDSC, underscoring the need for standardized pipelines, metadata, and rigorous batch correction methods [65,66,67]. Generalizability also remains a concern: models trained on immortalized cell lines may not fully capture the complexity of primary tumors. Validation in independent cohorts—whether PDO, PDX, or clinical datasets—is essential to ensure transportability and reduce lineage bias. Furthermore, pooled assays such as PRISM, while efficient, are prone to technical artifacts related to barcode skew, growth-rate differences, and plate effects; hits thus require orthogonal confirmation in arrayed or independent assays [68]. Finally, data harmonization and governance pose nontrivial barriers. Integrating heterogeneous omics modalities (e.g., RNA, protein, methylation, clinical data) demands complex preprocessing and normalization pipelines, and conclusions can vary depending on analytical choices. Moreover, privacy regulations and data-sharing constraints limit the linkage of clinical and omics data at scale, slowing external validation and clinical deployment [69]. Addressing these challenges will require continued development of transparent, reproducible, and ethically governed data infrastructures.

Clinical Application. The translational relevance of multi-omics pharmacogenomics is increasingly demonstrated across diverse cancer types. In Ewing sarcoma, dependency on the EWS–FLI1 fusion protein identified PARP inhibitor sensitivity as a biomarker-driven therapeutic vulnerability [64]. Baseline transcriptomic profiling has improved predictive accuracy for drug sensitivity relative to mutation-only models [63], while proteomic atlases spanning 949 cell lines have provided stable, protein-level predictors of drug response [8]. Practical translational workflows now apply pharmacogenomic models trained on cell-line datasets to patient tumors, predicting chemotherapy or targeted therapy response after batch harmonization with clinical RNA data [61,62]. Incorporation of proteomic features further stabilizes predictions and highlights actionable biomarkers that RNA alone may overlook [8,58]. Mechanism-aware repositioning has also become increasingly systematic: PRISM identified selective anticancer activity among non-oncology drugs with interpretable molecular correlates [13], and CMap/L1000 enables rational pairing of disease and drug signatures to propose resistance-reversal strategies or synergistic combinations [57]. Integration of dependency maps with pharmacologic data enables prioritization of actionable biomarkers and synthetic-lethal partners for basket and umbrella trial designs [59]. Translational pipelines increasingly follow a discovery-to-validation continuum—beginning with large-scale in vitro screening (e.g., CCLE, GDSC, PRISM, followed by orthogonal validation in PDO or ex vivo tissue models that preserve tumor microenvironmental features, retrospective testing in annotated clinical cohorts, and finally, prospective evaluation in biomarker-enriched clinical trials. Finally, the clinical implementation of pharmacogenomic prediction benefits from real-world emulation and continuous recalibration. Observational validations that emulate target trials help mitigate confounding when comparing outcomes of AI-guided versus standard-of-care selection strategies [69]. Co-clinical approaches that pair patient testing with PDO or PDX validation tighten mechanistic interpretation and reduce translational risk. As data governance improves, secure linkage of electronic health records and multi-omics repositories will transform pharmacogenomics into a continuously learning system, enabling dynamic model refinement and real-time decision support.

In summary, large-scale multi-omics integration has transitioned from retrospective discovery to prospective utility. By fusing orthogonal molecular, functional, and perturbational evidence, it is redefining biomarker discovery, drug repositioning, and clinical trial stratification. Sustained clinical adoption will depend on reproducible pipelines, robust external validation, and ethical governance that securely connects real-world clinical data with multi-omics predictions at scale [65,66].

## 6. Computational Drug-Screening Approaches: AI-Based Methods and Classical In Silico Modeling

Building on the multi-omics integration and data-driven pharmacogenomic principles outlined in Section 5, Section 6 focuses on the advanced computational models and in silico frameworks that operationalize these datasets into predictive, mechanistic, and clinically actionable drug-screening tools.

**Figure 3:**
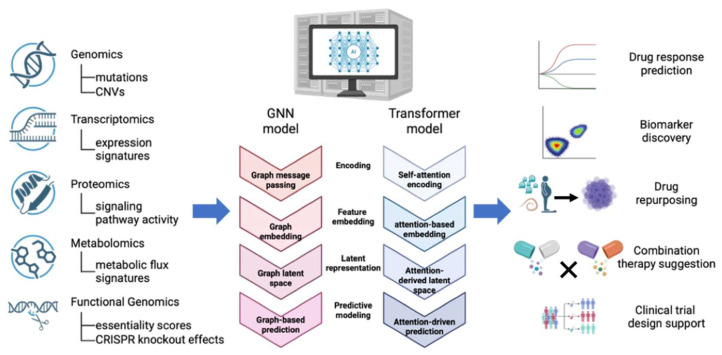
Integration of multi-omics data with artificial intelligence for precision oncology. Heterogeneous molecular datasets—including mutations and CNVs (genomics), expression signatures (transcriptomics), signaling-pathway activity (proteomics), metabolic-flux features (metabolomics), and CRISPR-derived essentiality scores (functional genomics)—are processed through model-specific encoding steps: graph message passing for GNNs and self-attention encoding for Transformers. These inputs are subsequently transformed into graph-based or attention-based embeddings, integrated into graph-structured or attention-derived latent spaces, and utilized in graph-based or attention-driven predictive modeling.

Principle. AI-based approaches and classical in silico modeling represent two distinct computational paradigms. AI and machine learning have become increasingly central to oncology research, integrating multi-omics, imaging, and chemical descriptors to accelerate drug discovery and biomarker identification. Figure 3 conceptually summarizes how AI frameworks synthesize heterogeneous molecular layers into clinically actionable insights, including drug response prediction, biomarker discovery, and rational therapy design. AI models encompass a wide spectrum—from traditional linear regression to deep neural networks, graph convolutional architectures for molecular structures, and generative adversarial networks for de novo compound design [14,70,71]. In silico drug screening —traditionally referring to physics-based, rule-based, or simulation-driven computational models—leverages patient-derived genomic, proteomic, and phenotypic data, along with chemical structure features, to predict therapeutic responses using advanced computational frameworks. These systems integrate multi-omics inputs—mutations, gene expression, proteomics, epigenomics—and clinical data into unified predictive models [72,73]. For example, the Multi-Omics Machine Learning Integrative Network (MOMLIN) framework jointly analyzes mutation, expression, and tumor microenvironment profiles alongside clinical data, achieving near-perfect accuracy in distinguishing responders from non-responders in breast cancer [72]. Similarly, transformer-based “foundation” models pre-trained on large cell-line compendia (e.g., CCLE, GDSC, PRISM) can be fine-tuned on limited PDO or cohort datasets to enhance generalizability [73]. Generative deep-learning methods such as variational autoencoders or generative adversarial networks (GANs) can further explore vast chemical space, proposing novel drug-like molecules guided by learned bioactivity patterns [74]. In essence, AI approaches now span from graph neural networks that encode molecular and biological network information to reinforcement-learning systems that iteratively optimize compounds for efficacy and ADMET (absorption, distribution, metabolism, excretion, and toxicity) properties—all designed to accelerate hypothesis generation and lead identification beyond traditional docking or rule-based screening [73,74].

Together, these AI-derived molecular representations enable key predictive applications, including drug-response modeling, biomarker discovery, drug-repurposing candidate prioritization, rational combination-therapy suggestion, and support for molecularly informed clinical-trial design.

Advantages. AI-driven drug discovery and screening offer multiple advantages that extend well beyond classical computational methods. The foremost is scalability: these models can evaluate millions of chemical structures or thousands of patient-derived datasets simultaneously, drastically reducing the time and cost associated with experimental screening. By prioritizing high-probability candidates, AI systems significantly improve the cost-efficiency of early-stage discovery pipelines. A second advantage lies in complex pattern recognition. Machine-learning models excel at capturing nonlinear relationships among omics, imaging, and clinical variables that would be invisible to linear statistical approaches. Integrative frameworks that merge genomics, transcriptomics, proteomics, and other omics layers within a single predictive model often outperform single-omic analyses by uncovering synergistic biomarker signals. For instance, MOMLIN identified interconnected mutation–expression networks that enhanced predictive power for drug response in cancer [72]. AI also enables scalable exploration of chemical space. Generative algorithms such as GANs can learn from known molecules to propose billions of plausible variants, effectively mapping the landscape of potential drug-like compounds [74]. This capability accelerates hit discovery by several orders of magnitude compared to classical docking workflows. A further strength is adaptivity through transfer learning. Because real-world clinical data are limited, many models are pre-trained on extensive pharmacogenomic datasets and subsequently fine-tuned on smaller organoid or patient cohorts to improve generalization. For example, the PharmaFormer model, pre-trained on pan-cancer transcriptomic and drug-sensitivity datasets, was fine-tuned using tumor organoid data, markedly improving clinical response prediction across multiple tumor types [73]. AI frameworks can also integrate real-world data such as electronic health records (EHRs), registries, and radiology/pathology reports. The MSK-CHORD initiative, for instance, harmonized EHR-derived notes, medication histories, and genomic data for many patients, uncovering novel predictors of metastasis and survival [75]. Incorporating real world data into predictive models helps bridge preclinical insights and clinical applicability by grounding in silico predictions in observed treatment outcomes. Finally, AI supports interpretable hypothesis generation. Modern explainable-AI methods quantify the contribution of each feature—mutation, pathway, or clinical variable—to a prediction, revealing mechanistic hypotheses and guiding experimental validation [76,77]. Such interpretability transforms AI from a purely predictive tool into a discovery engine capable of identifying synthetic-lethal interactions or unanticipated drug synergies [72,74].

Limitations. Despite rapid progress, substantial barriers limit the reliability, generalizability, and clinical readiness of AI-based drug–response prediction. A fundamental challenge is domain shift: models trained on controlled experimental systems often fail when confronted with the biological and technical heterogeneity of real-world data. Even state-of-the-art models optimized on CCLE, GDSC, or PRISM frequently lose accuracy when applied to PDOs, PDXs, or clinical specimens, reflecting profound differences in stromal composition, immune context, data-generation pipelines, and therapeutic exposure. Existing domain-adaptation approaches only partially mitigate these gaps, and cross-platform reproducibility remains the exception rather than the rule [78,79]. Bias and data imbalance introduce an equally serious limitation. Most training datasets disproportionately represent common tumor types, cell lines with high proliferative capacity, or patients from specific demographic groups. As a consequence, AI models may learn spurious correlates rather than true biological determinants of drug response. Without explicit bias audits and stratified external validation, these systems risk entrenching or amplifying disparities in therapeutic prediction [80,81]. A further, and often underappreciated, issue is poor reproducibility and lack of methodological transparency. Performance can vary drastically with different preprocessing pipelines, hyperparameter settings, batch-correction strategies, or random seeds. Many published models are not accompanied by fully reproducible code, version control, or detailed documentation, making independent replication nearly impossible [82]. Multi-omics integration especially suffers from irreproducible feature engineering choices, undermining confidence in mechanistic interpretation [83]. Interpretability remains limited: deep neural networks often provide predictions without biologically meaningful explanations, impeding clinical trust and hindering regulatory review. Although explainable-AI frameworks are available, their outputs often reflect post hoc descriptions of model behavior rather than meaningful mechanistic insight into the underlying biology [84]. Finally, regulatory, ethical, and governance barriers pose major hurdles. Current frameworks were designed for static diagnostic algorithms, not adaptive models that update as new data are encountered. Requirements for data provenance, model auditing, and Good Machine Learning Practices (GMLP) make widespread clinical deployment challenging. Privacy restrictions and fragmented data infrastructures further prevent large-scale, multi-institutional validation, delaying translation from proof-of-concept to practice. Collectively, these challenges highlight that AI in drug screening is advancing rapidly but remains far from ready for unqualified clinical adoption. Overcoming these issues will require rigorous external validation, transparent methodologies, comprehensive bias mitigation, and harmonized regulatory pathways.

Clinical Application. AI-based drug screening has begun to transition from retrospective proof-of-concept toward prospective clinical implementation. Models trained on large pharmacogenomic datasets and fine-tuned with limited PDO data have achieved high accuracy in predicting clinical treatment responses [14]. Integration with organoid platforms allows adaptive, iterative testing, where AI systems propose drug combinations for experimental validation in near-real time. Beyond molecular data, AI applied to imaging has proven valuable for treatment stratification. Deep-learning histopathology models can classify tumor subtypes and predict therapeutic response, complementing molecular assays and drug-sensitivity data [71]. In parallel, in silico frameworks have expanded beyond tumor-intrinsic predictions to encompass systemic pharmacology, including ADMET and PK/PD modeling. Modern machine-learning systems can estimate absorption, distribution, metabolism, excretion, and toxicity profiles directly from chemical structure, physicochemical descriptors, and multi-omics context, enabling early elimination of compounds with poor drug-likeness or safety liabilities [85,86]. Deep-learning toxicology models trained on large chemoinformatic datasets now achieve high accuracy in predicting cardiotoxicity, hepatotoxicity, and off-target interactions, while AI-based PK/PD models integrate predicted systemic exposure with dynamic biological response to support dose optimization and therapeutic-window estimation [86,87]. These computational approaches complement tumor-centric drug-screening platforms by evaluating systemic feasibility and translational readiness, thereby improving the efficiency and safety of early-stage drug development. Regulatory agencies have begun to recognize AI-assisted evidence, although full acceptance still depends on transparent validation and reproducible performance. A recent multi-institutional study exemplifies the potential of AI-guided treatment selection: Saad et al. analyzed clinicogenomic data from approximately 2300 patients with metastatic non-small-cell lung (NSCLC) cancer across four institutions [88]. The resulting A-STEP model, trained on 28 genomic and clinical features, predicted which patients benefited from adding chemotherapy to PD-1/PD-L1 inhibitors. Retrospective analysis showed that patients treated according to the AI’s recommendation experienced significantly better progression-free survival than those treated by standard biomarker rules [88]. AI is also beginning to influence trial design. Early “AI-augmented” precision-oncology trials now incorporate ML modules that analyze longitudinal tumor genomics to suggest therapy adjustments during the trial. Efforts such as WINTHER and I-PREDICT, which match therapies to multi-omics tumor profiles, are integrating AI prioritization modules to guide drug selection, with results pending. Moreover, learning health-system initiatives that continuously retrain models on new patient data are enabling dynamic, data-driven adaptive trial designs.

In summary, AI-based drug screening and prediction are rapidly evolving from retrospective validation to prospective, trial-integrated applications. The first wave of implementations focuses on augmenting clinician decision-making with model-based recommendations, followed by real-time adaptation within ongoing trials. Looking ahead, fully autonomous AI-guided screening pipelines could eventually support continuous learning and adaptive therapy assignment. However, widespread clinical acceptance will depend on rigorous external validation, transparent reporting, and alignment with emerging regulatory frameworks.

## 7. Drug Repositioning in Cancer: Case Studies

Rationale. Drug repositioning, or repurposing, leverages the established safety profiles and PK of approved drugs to accelerate therapeutic development. In oncology, this approach is particularly attractive given the high attrition rates and lengthy timelines associated with de novo drug discovery. Recent analyses estimate that a repositioned agent can reach regulatory approval in approximately 6.5 years with an investment of ~US $300 million, compared with 13–15 years and $2–3 billion for a novel oncology drug [89,90]. Because these compounds have already passed Phase I safety testing, extensive data on toxicity, dosing, and PK are available, thereby reducing risk in later-stage trials [89]. Notably, repurposed drugs tend to fail less frequently overall than new molecular entities. These pharmaco-economic advantages have motivated dedicated initiatives, including the NIH NCATS program (a US translational science initiative promoting new therapeutic uses for existing drugs), the Wellcome Trust’s global funding efforts, the Broad Institute’s Drug Repurposing Hub (a comprehensive database of approved and investigational compounds), and non-profit consortia such as ReDO (Repurposing Drugs in Oncology), which coordinates international efforts to evaluate off-patent drugs in cancer therapy [91]. However, repositioning also faces structural and ethical challenges. Many candidate agents are off-patent generics, limiting industry incentives to finance costly trials and commercialization. Multi-stakeholder collaborations between academia, public agencies, and non-profit organizations are therefore often required. Ethical issues may also arise when clinicians or patients pursue off-label use before rigorous evidence is available, emphasizing the need for transparent consent and data disclosure. Conversely, validated repositioned therapies could greatly enhance global access because generic drugs are inexpensive and widely distributed. Still, pharmacologic and tissue-specific differences often limit success: effective anticancer doses may exceed those used for the original indication, and in many cases achievable concentrations are sub-therapeutic. Consequently, formulation improvements or combination strategies are frequently necessary. In summary, while repositioning offers clear economic and developmental advantages [89,90,91], its translational success ultimately depends on novel funding frameworks, biomarker-driven clinical trials, and equitable implementation.

This section provides an integrated mechanistic overview of the repurposed agents discussed in this section. Table 3 shows a summary table that compares their proposed mechanisms, clinical outcomes, and mechanistic reasons for failure, suggesting the key translational lessons across these case studies.

### 7.1. Metformin

Metformin, a widely used treatment for type 2 diabetes, was hypothesized to exert anticancer effects via AMPK activation, mTOR inhibition, and metabolic reprogramming. Observational studies suggested lower cancer incidence and improved survival among diabetic patients receiving metformin [92,93,94]. However, the large randomized MA.32 trial in breast cancer failed to demonstrate any survival benefit, and among ~3600 patients, the addition of metformin to standard therapy produced no difference in invasive disease-free survival [95]. These findings illustrate how observational associations can be confounded by comorbidities such as obesity. Mechanistically, metformin’s modulation of cellular metabolism remains compelling, yet its therapeutic benefit may depend on specific molecular contexts or synergistic combinations rather than broad unselected use. Importantly, several PK studies have shown that the intratumoral concentrations required for AMPK activation or mTOR suppression are far higher than those achievable with clinically tolerated dosing, suggesting a fundamental PK/PD mismatch.

### 7.2. Hydroxychloroquine (HCQ)

Originally developed as an antimalarial and later repurposed for autoimmune diseases, hydroxychloroquine functions as an autophagy inhibitor. It has been evaluated in pancreatic cancer as a chemosensitizer, with early-phase studies showing enhanced pathological responses but no survival improvement in larger cohorts [96]. HCQ raises lysosomal pH and blocks late-stage autophagy, but this inhibition is often incomplete at clinically tolerable doses. Consequently, subsequent efforts have focused on more potent autophagy inhibitors or rational combinations (e.g., HCQ with mTOR or ERK inhibitors). To date, HCQ alone has not demonstrated a definitive clinical benefit. These disappointing results likely arose from incomplete autophagy inhibition at tolerable doses. Preclinical models typically require HCQ concentrations well above the maximum feasible human exposure to fully block lysosomal function, and further dose escalation is limited by QT prolongation and gastrointestinal toxicity. Thus, the negative trial outcomes may reflect a PK-limited failure of target engagement rather than a lack of biological rationale.

### 7.3. Statins

Statins, HMG-CoA reductase inhibitors, have long been proposed as anticancer agents owing to their pleiotropic effects on cell signaling and RAS prenylation. Preclinical studies showed variable tumor-suppressive activity, but clinical results have been inconsistent. Meta-analyses of randomized trials indicate that statins do not reduce overall cancer incidence or mortality [97,98,99,100]. Although mechanistically sound, their anticancer impact appears modest and potentially restricted to specific molecular subsets. Statins therefore exemplify pleiotropic agents whose efficacy may depend on precise patient selection or combination with targeted therapies. A major limitation of prior trials is that they enrolled unselected patient populations, despite evidence that only tumors with high mevalonate-pathway dependence or defective prenylation feedback are statin-sensitive. Without molecular stratification, any true subset benefit would have been diluted, contributing to the null results observed in randomized studies.

### 7.4. Mebendazole (MBZ)

Mebendazole, an anti-helminthic drug that binds β-tubulin and inhibits microtubule polymerization, has shown cytotoxic activity in preclinical models—particularly glioblastoma [101]. Despite encouraging in vitro data, clinical trials failed to demonstrate survival benefit, primarily due to poor bioavailability (especially within the CNS) and a narrow therapeutic window. The gap between laboratory potency and achievable systemic exposure underscores pharmacokinetic limitations as a recurring barrier in repurposing. In glioblastoma, therapeutic failure is largely attributable to inadequate CNS penetration; MBZ achieves only minimal brain concentrations relative to the nanomolar potency observed in vitro. Additionally, its narrow therapeutic window precludes dose escalation to overcome this barrier, illustrating how tissue-specific PK constraints can negate promising preclinical activity.

### 7.5. Thalidomide

Thalidomide represents one of the most notable successes in biomarker-guided drug repurposing [102]. Originally developed as a sedative, it demonstrated profound antitumor activity in multiple myeloma once its mechanism of action was linked to cereblon (CRBN), a substrate receptor of the CRL4 ubiquitin ligase complex [103]. CRBN binding induces neomorphic degradation of the transcription factors IKZF1 and IKZF3—critical dependencies in myeloma—thereby producing highly selective cytotoxicity [104]. This mechanistic insight enabled the rational development of next-generation Immunomodulatory Drugs, including lenalidomide and pomalidomide, which have transformed myeloma therapy. Thalidomide thus illustrates that when molecular determinants of sensitivity are well defined, repositioning can achieve durable clinical success.

### 7.6. Other Candidates and Data-Driven Pipelines

Beyond individual drugs, numerous repositioning candidates—such as aspirin, NSAIDs, and angiotensin-pathway inhibitors—have exhibited modest or subgroup-specific effects without altering clinical practice. Increasingly, modern repurposing relies on large-scale omics and AI-based approaches to generate mechanism-driven hypotheses. The LINCS/Connectivity Map (CMap) project, for instance, has compiled gene-expression signatures for numerous drug perturbations, enabling systematic matching between disease and drug profiles [105,106,107]. The DepMap/PRISM initiative tested ~4500 drugs—including many non-oncology agents—across 578 cell lines, identifying selective vulnerabilities such as the alcohol-aversion drug disulfiram being cytotoxic to cells with low metallothionein expression, or the NSAID tepoxalin targeting cells with high ABCB1 expression [13,108]. In parallel, graph-based AI models like TxGNN have demonstrated improved ability to predict new drug–disease indications across thousands of conditions [13,109]. Collectively, these efforts represent a shift from serendipitous discovery toward data-driven repurposing pipelines, though all computational predictions still require rigorous experimental validation.

Lessons Learned. Several overarching lessons have emerged from the history of oncology drug repurposing. First, positive preclinical or observational findings alone are insufficient; biomarker-driven trial designs are essential for success. More broadly, many repositioning failures stem not from invalid biological hypotheses but from failures in pharmacology or patient selection. For example, several agents—such as metformin, statins, and hydroxychloroquine—were evaluated in unselected populations, despite evidence that only molecularly defined subgroups (e.g., AMPK-high, mevalonate-dependent, or autophagy-addicted tumors) may benefit. In parallel, PK/PD constraints frequently prevented adequate target engagement; clinically tolerable doses of metformin or hydroxychloroquine fall short of the intratumoral concentrations required for pathway inhibition, while mebendazole fails to achieve therapeutic CNS levels. Thus, both prospective molecular stratification and rigorous exposure–response modeling are indispensable components of successful repurposing strategies. The growing use of real-world data is a double-edged sword: analyses of EHR and registry data can highlight potential associations between approved drugs and cancer outcomes, but most studies suffer from fragmented data, uncontrolled confounding, and lack of causal inference [110]. Therefore, signals derived from real-world data should be considered hypothesis-generating rather than confirmatory, as observational associations often lack causal inference. Second, many repositioned drugs exhibit limited potency or specificity in tumor contexts. Doses effective in the original indication may be too low for anticancer efficacy, while escalation is constrained by toxicity. For example, hydroxychloroquine dose escalation in pancreatic cancer was limited by side effects, likely preventing complete autophagy inhibition. Consequently, most repurposed agents display only modest single-agent activity, reinforcing the need for combination regimens or optimized formulations. Third, success will increasingly depend on molecular stratification. Integrating genomics, proteomics, and metabolomics enables the identification of tumor subgroups most likely to respond to specific repurposed agents. The PRISM screen, for instance, revealed that sensitivity to certain compounds correlates with expression of specific biomarkers—such as SLFN12 dependency for PDE3A-binding drugs [13,111,112,113,114]. Adaptive and basket trial designs, which enroll patients based on molecular rather than anatomical tumor characteristics, are particularly suited to such strategies. Finally, transparency regarding both positive and negative outcomes is critical. Learning from failure—understanding when and why a repurposed agent did not succeed—is as informative as celebrating successes. Collectively, these lessons underscore that repurposing is not a shortcut around rigorous clinical validation; it is a scientifically grounded translational enterprise that must combine biological insight, precision stratification, and well-designed clinical evaluation to realize its full potential.

## 8. Translating Drug Screening to Clinical Trials

Principle. Ultimately, the value of preclinical screening lies in its translation to patient benefit. Modern clinical trial designs—particularly basket and umbrella trials—enable efficient evaluation of biomarker-guided therapies, bridging the gap between laboratory discoveries and real-world precision oncology.

### 8.1. Basket Trials

Basket trials evaluate a single targeted therapy across multiple tumor origins that share a common molecular alteration. This “treat different cancers with the same drug” paradigm exemplifies the tissue-agnostic approach to precision oncology. In such trials, patients with any type of cancer are eligible if their tumors harbor the relevant biomarker. A landmark example is the TRK inhibitor, larotrectinib, which achieved durable responses across both pediatric and adult malignancies harboring NTRK gene fusions, leading to one of the first tissue-agnostic FDA approvals [115]. Similarly, pembrolizumab received approval in 2017 for any microsatellite-instability-high (MSI-H) or mismatch-repair–deficient (dMMR) solid tumor, regardless of histology [116,117]. These successes established basket designs as a foundation for pan-cancer indications, later extended to other targets such as NTRK fusions (entrectinib) and BRAF^V600E^ mutations (dabrafenib + trametinib) [115,116,118]. At the same time, basket trials can uncover histology-specific resistance. In a pan-cancer BRAF basket study, vemurafenib showed marked activity in BRAF-mutant lung and other tumors but not in colorectal cancer, revealing lineage-dependent signaling differences [115,119]. Such results often motivate follow-up, tissue-specific studies. Large adaptive baskets such as NCI-MATCH illustrate the scalability of this framework. NCI-MATCH matches patients with diverse tumor types to molecularly defined treatment arms, dynamically adding or closing cohorts as new targets or agents emerge [116,120]. These studies concentrate patients with rare alterations, thereby improving power to detect meaningful responses, but they also require stringent biomarker validation and reliable companion diagnostics. Accordingly, the FDA continues to refine its policies on biomarker testing and companion diagnostic (CDx) development to ensure that enrolled patients indeed possess validated molecular targets [116].

### 8.2. Umbrella Trials

In contrast, umbrella trials test multiple targeted therapies within a single cancer type, stratifying participants according to their tumor’s molecular features. This design embodies the concept of “treating the same disease with different treatments.” For instance, in NSCLC, patients may be screened for EGFR mutations, ALK or ROS1 fusions, or KRAS and BRAF mutations and then assigned to the appropriate targeted inhibitor [116]. Landmark umbrella studies such as Lung-MAP (in squamous NSCLC) and the National Lung Matrix Trial (in adenocarcinoma) exemplify this adaptive design [121,122,123]. Typically, umbrella protocols incorporate a shared control arm, whereby biomarker-negative patients receive standard chemotherapy or immunotherapy. The FDA has encouraged such shared-control structures to improve trial efficiency and ensure that all participants receive evidence-based care [116]. Because umbrella trials focus on a single cancer type, they allow direct comparison of multiple targeted strategies under uniform clinical conditions. Adaptive features—such as response-based arm expansion or early closure—permit ongoing optimization. However, these trials also face statistical and logistical challenges: rare subgroups may accrue slowly, overlapping biomarkers require prespecified assignment rules, and multiple-hypothesis correction must be carefully controlled. When executed successfully, umbrella trials enable individualized therapy selection within one cancer type while generating comparative efficacy data across regimens [116].

### 8.3. Regulatory Context

Regulatory agencies now actively support these innovative frameworks. The U.S. FDA issued 2018 guidance endorsing master protocol designs—including basket, umbrella, and platform trials—as tools to accelerate oncology drug development [115]. Under this guidance, any biomarker used for trial stratification must undergo rigorous analytical and clinical validation prior to patient enrollment. Companion diagnostics (CDx) are integral to this process and are increasingly required for targeted therapy approvals. Examples include PCR-based assays for EGFR^T790M^ mutations in osimertinib treatment of NSCLC, and IVD-based testing for HER2, BRAF, or ALK alterations [116,124]. As of 2025, the FDA lists more than 150 cleared or approved CDx devices [125]. These regulatory expectations ensure that trial populations are molecularly defined and that test performance meets reproducibility and accuracy standards. Comparable policies in Europe and Asia likewise mandate that only patients with verified molecular targets are enrolled, reinforcing patient safety and interpretability of biomarker-driven results [116].

### 8.4. Integration with Preclinical Screening

Emerging data integration strategies now link preclinical screening platforms—such as PDO, PDX, and organ-on-chip system—to clinical trial design. These translational bridges help identify biomarkers and stratification strategies before patient enrollment. For example, co-clinical trial approaches simultaneously treat patient cohorts and matched preclinical models, allowing iterative refinement of hypotheses [126]. A recent “Phase II–like” screen of 49 head-and-neck cancer PDX models identified novel biomarkers of cetuximab resistance—including ANKH amplification and PARP3 overexpression—and even proposed rational combination therapies to overcome resistance [127]. Similarly, PDOs preserve the histological and genomic characteristics of primary tumors [24], and ex vivo drug sensitivity in PDO panels has been shown to predict patient outcomes [23]. Large-scale pharmacogenomic resources such as DepMap and PRISM further complement these efforts by correlating genomic alterations with drug responses, thereby nominating biomarker–drug pairs suitable for basket or umbrella testing. Computational analysis of dependency maps can highlight synthetic-lethal relationships and prioritize trial arms for validation. In practice, feedback between preclinical and clinical studies is increasingly iterative: ineffective arms can be deprioritized early, while unexpected sensitivity signals in models can prompt new cohorts. Selecting patients whose tumors—or matched models—demonstrate drug responsiveness in preclinical systems enhances the likelihood of clinical success. Ultimately, this integrative cycle between laboratory screening and biomarker-driven trial design accelerates the translation of experimental findings into effective, patient-specific therapies.

## 9. Future Directions

Looking ahead, the field of cancer drug screening is rapidly converging toward models and frameworks that are not only more predictive of human biology but also more ethically and computationally sustainable. A key development in this transition is the growing regulatory acceptance of human-relevant and non-animal testing methodologies. The U.S. FDA Modernization Act 2.0, enacted in 2022, marks a paradigm shift by formally allowing alternative approaches—including organoids, microphysiological systems, and computational models—to substitute for certain animal studies in drug development [128,129]. In parallel, frameworks to assess the scientific reliability of these New Approach Methodologies are emerging. Van der Zalm et al. proposed a Scientific Confidence Framework emphasizing human biological relevance, technical robustness, and data transparency as core criteria for regulatory adoption [130]. Together, these initiatives outline a clear trajectory toward mechanism-based, human-centric validation strategies that could ultimately redefine preclinical standards.

Ethical and governance considerations will be equally central to this transformation. The expansion of PDO and PDX biobanking requires transparent consent processes, robust oversight, and protection of patient privacy [131,132]. Ensuring equitable access to these advanced technologies is essential to prevent the widening of global disparities in research participation and therapeutic benefit. Meanwhile, the deployment of AI models in screening and clinical decision-making demands explainability and continuous auditing to mitigate algorithmic bias and preserve clinician and patient trust [133].

Another frontier lies in the integration of preclinical screening with real-world clinical data. Linking drug-response datasets with EHRs and real-world evidence enables a continuous learning feedback loop between bench and bedside [134]. Federated-learning infrastructures now permit collaborative model building across institutions without direct data exchange, safeguarding privacy while dramatically expanding sample diversity [135,136]. In parallel, model fidelity continues to improve through next-generation platforms: immune-enhanced PDO incorporating autologous immune cells or stromal components [137,138,139]; organ-on-a-chip hybrids that combine 3D structure with microfluidic perfusion [140]; and humanized PDX embedding patient-specific immune systems to evaluate immunotherapy responses [141,142]. Isogenic organoid series further enable causal interrogation of specific mutations [143,144], while metastatic-niche models replicate tumor–host interactions within secondary organs to study colonization dynamics [145].

Realizing the translational potential of these innovations will require unprecedented collaboration and standardization. Multi-center “ring” studies—collaborative inter-laboratory experiments in which identical assays are independently repeated across multiple sites—are essential to ensure reproducibility and standardization [146]. Shared standard-operating procedures, reference materials, and global consortia will be indispensable for achieving regulatory recognition [147]. Finally, closer integration of academic and biopharmaceutical pipelines can accelerate the progression from preclinical validation to clinical impact [148]. Collectively, these directions underscore a future in which drug screening evolves from isolated experimental systems into an interconnected, ethically grounded, and computationally adaptive network that continually refines itself through real-world feedback and cross-sector collaboration.

## Figures and Tables

**Table 1:** Comparative features of major preclinical cancer drug screening models.

Model	Cost	Time to Establish/Test	Patient Correlation (Genetics/Histology/Response)	Immune System Representation	Throughput	Typical Applications
2D Cell Lines	Low	Days–weeks	Low (adapted, clonal, lacking microenvironment)	Absent	Very high (HTS compatible)	Large-scale screening, pharmacogenomics databases (NCI-60, CCLE, GDSC)
PDOs	Moderate	1–3 weeks (establishment), expandable	High (retain tumor genetics and heterogeneity)	Limited (epithelial cells only; immune/stroma usually absent)	High (multi-well, automation possible)	Personalized therapy testing, drug mechanism studies
PDXs	Very high	2–6 months (engraftment and expansion)	High (preserve histology, clonal architecture, in vivo drug response)	Partial (mouse stroma; human immunity absent unless humanized)	Low (few drugs per patient due to cost/time)	Co-clinical trials, in vivo validation, PK
Organ-on-a-Chip Systems	High (specialized devices)	Days–weeks (chip seeding and stabilization)	Moderate–High (microenvironment cues and fluid flow replicated)	Limited (some stromal/immune co-culture possible)	Low–Moderate (currently small scale, improving with automation)	Mechanistic studies, toxicity evaluation, multi-organ interaction, precision oncology
In Silico Models	Low–Moderate (computational resources)	Hours–days	Variable (depends on training data quality and patient alignment)	N/A	Very high (screen millions of compounds virtually)	Virtual screening, docking studies, PK/PD simulations

2D: two-dimensional, PD: pharmacodynamics, PDO: patient-derived organoid, PDX: patient-derived xenograft, PK: pharmacokinetics, HTS: high-throughput screening, NCI-60: National Cancer Institute-60 cell line panel, CCLE: Cancer Cell Line Encyclopedia, GDSC: Genomics of Drug Sensitivity in Cancer.

**Table 2:** Comparative diagnostic performance of PDO and PDX models across major tumor types.

Tumor Type	Sensitivity		Specificity		Concordance (Overall Agreement)	
	PDO	PDX	PDO	PDX	PDO	PDX
Esophageal [29,30,31]	100%	28–87%	93%	58%	~70%	71%
Gastric [30,31]	100%	96%	93%	70%	~95%	~70%
Colorectal [32,33,34,35,36,37]	84–100%	96%	92–93%	70%	70–90%	64–85%
Hepatocellular [29]	~85%	87%	~60%	58%	~70%	~71%
Pancreatic [37,38,39]	83.3%	96%	92.9%	70%	~85%	~87%
Lung [37,39,40]	84%	96%	83%	70%	83%	~87%
Breast [29,34,41]	Not reported	100% (in small TNBC cohort)	Not reported	100%	70–77%	70–76%
Ovarian [42,43]	100%	100%	100%	100%	100%	100%

PDO: patient-derived organoid, PDX: patient-derived xenograft.

**Table 3:** Mechanistic Summary of Repositioned Drugs in Oncology: Proposed Mechanisms, Clinical Outcomes, and Reasons for Failure.

Drug	Cancer Type	Mechanism	Study Type	Clinical Outcome	Mechanistic/Translational Reason for Failure
Metformin	Breast cancer	AMPK activation, mTOR inhibition, metabolic reprogramming	Phase III RCT in early-stage breast cancer	no improvement in invasive disease–free survival	PK/PD mismatch (intratumoral concentrations too low); no biomarker stratification; confounding in observational studies.
Hydroxychloroquine	Pancreatic cancer	Autophagy inhibition via lysosomal pH elevation, blockade of autophagosome–lysosome fusion	Phase II neoadjuvant trial	early-phase studies showed improved pathological response, but no survival benefit	Incomplete autophagy inhibition; required concentrations not clinically achievable; QT/GI toxicities; insufficient target engagement.
Statins	Multiple cancer type	HMG-CoA reductase inhibition; suppression of mevalonate pathway; impaired RAS prenylation	Not Clinical trial (Meta-analysis)	no consistent improvement in incidence or mortality	Unselected populations; benefit limited to mevalonate-dependent tumors; pleiotropic effects dilute efficacy; no biomarker-driven stratification.
Mebendazole	Glioblastoma	β-tubulin binding; inhibition of microtubule polymerization	Phase I/II early-phase trials	no significant clinical benefit	Poor CNS penetration; sub-therapeutic brain levels; limited dose escalation; PK constraints overriding potent in vitro activity.
Thalidomide (successful case)	Multiple myeloma	CRBN-mediated neomorphic degradation of IKZF1/IKZF3	Phase II/III clinical trials in relapsed/refractory and newly diagnosed multiple myeloma	Major clinical success; substantial improvements in response rates and survival	Defined CRBN dependency; biomarker-aligned disease biology; strong target engagement; foundation for next-generation IMiDs.

AMPK: AMP-activated protein kinase, mTOR: mechanistic target of rapamycin, QT: Interval from the Q wave to the T wave, GI: gastrointestinal, HMG-CoA: 3-hydroxy-3-methylglutaryl–coenzyme A, CNS: central nervous system, CRBN: cereblon, IKZF1/3: IKAROS family zinc-finger proteins 1 and 3, IMiD: immunomodulatory drug.

## Data Availability

No new data were created or analyzed in this study. Data sharing is not applicable to this article.

## Abbreviations

The following abbreviations are used in this manuscript:

2D	Two-dimensional
3D	three-dimensional
AI	artificial intelligence
AUC	area under the curve
CCLE	Cancer Cell Line Encyclopedia
CDx	companion diagnostic
CMap	Connectivity Map
DepMap	Dependency Map
dMMR	mismatch-repair–deficient
EHRs	electronic health records
GDSC	Genomics of Drug Sensitivity in Cancer
GANs	generative adversarial networks
HCQ	Hydroxychloroquine
MBZ	Mebendazole
MSI-H	microsatellite-instability-high
NAMs	New Approach Methodologies
NSCLC	non–small-cell lung cancer
PD	Pharmacodynamics
PDO	patient-derived organoid
PDX	patient-derived xenograft
PK	Pharmacokinetics
ReDO	Repurposing Drugs in Oncology
RNAi	RNA interference
sgRNAs	small guide RNAs
shRNAs	short hairpin RNAs
